# Long anterior lens zonules with retinal stripes: a case report

**DOI:** 10.1186/s12886-022-02496-7

**Published:** 2022-06-23

**Authors:** Xiaolu Cao, Jiayin Qin, Zhiyong Li, Peipei Jia, Beibei Gao, Lin Feng, Wei Wang

**Affiliations:** 1 Hebei Ophthalmology Key Lab, Hebei Eye Hospital, No. 399, Quanbei East Street, Xingtai, 054000 Hebei China; 2 Department of Ophthalmology, Life Science Park of Zhong Guancun, Peking University International Hospital, Life Park Road No.1, Changping District, Beijing, 102206 China

**Keywords:** Long anterior lens zonules, Retinal stripes, High intraocular pressure, Case report

## Abstract

**Background:**

Long anterior lens zonules   (LAZs) is a rare disease that was mostly conducted among African Americans. Through the observation of a Chinese patient, we discoverd that the disease may show different characteristics in Asians.

**Case presentation:**

A patient with vision loss due to a macular hole was found to have several special clinical signs during vitrectomy combined with phacoemulsification and intraocular lens implantation surgery in our hospital, including radially oriented lines on the anterior capsule with pigment, a shallow anterior chamber, slightly high intraocular pressure, and radial retinal stripes in the peripheral retina. Finally, he was diagnosed with long anterior lens zonule syndrome.

**Conclusion:**

Clinicians need to pay more attention to the rare disease LAZs. It is important to tear the appropriate size of the anterior capsule so as to avoid radial capsular tearing and intraocular lens dislocation.

**Supplementary Information:**

The online version contains supplementary material available at 10.1186/s12886-022-02496-7.

## Background

Long anterior lens zonules (LAZs) are observed as fine, radially oriented lines on the anterior capsule of the crystalline lens during slit-lamp examination. Another phenotype has been reported in some literatures, in which LAZs are associated with late-onset retinal and macular degeneration (L-ORMD). It sometimes causing a marked reduction in the ZFZ diameter. The estimated prevalence is from 1–2% and is associated with age > 50 years, female sex, and most binocular diseases [1, 2]. But previous studies were mostly conducted among African Americans. Through the observation of a Chinese patient, we discoverd that the disease may show different characteristics in Asians.

## Case presentation

**Fig. 1:**
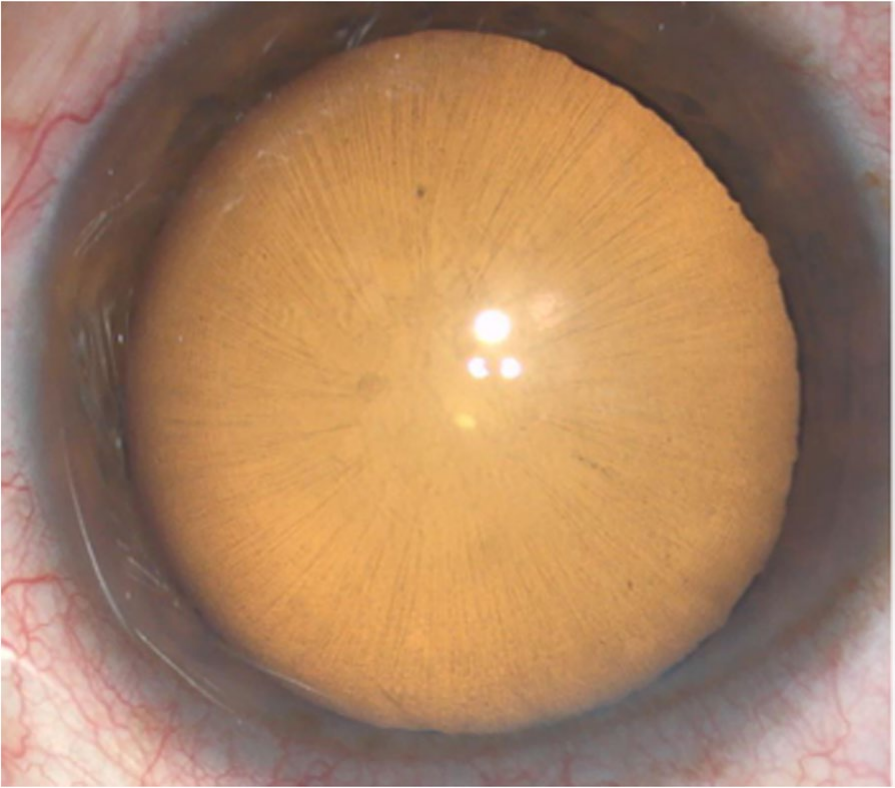
Pigmented, radially oriented lines on the anterior capsule were observed bilaterally, and were more obvious in the right eye than in the left

A 60-year-old Chinese woman presented with a two-month history of blurred vision and distortion. The patient had been diagnosed with hypertension for eight years and diabetes for two years, without limb or heart abnormalities. There was no history of trauma or eye surgery, and their family history was unremarkable. The best corrected visual acuity was 0.02 OD and 0. 6 OS, with intraocular pressure (IOP) of 23 mmHg OD and 21 mmHg OS. Slit-lamp microscopy showed a mild shallow anterior chamber of the right eye and normal anterior chamber depth of the left eye. Cataract was observed bilaterally, with a cloudy cortex and nucleus. Many pigmented, radially oriented lines on the anterior capsule were observed bilaterally, which were more obvious in the right eye than in the left eye (Fig. 1). Therefore, we diagnosed this patient as LAZs.

**Fig. 2:**
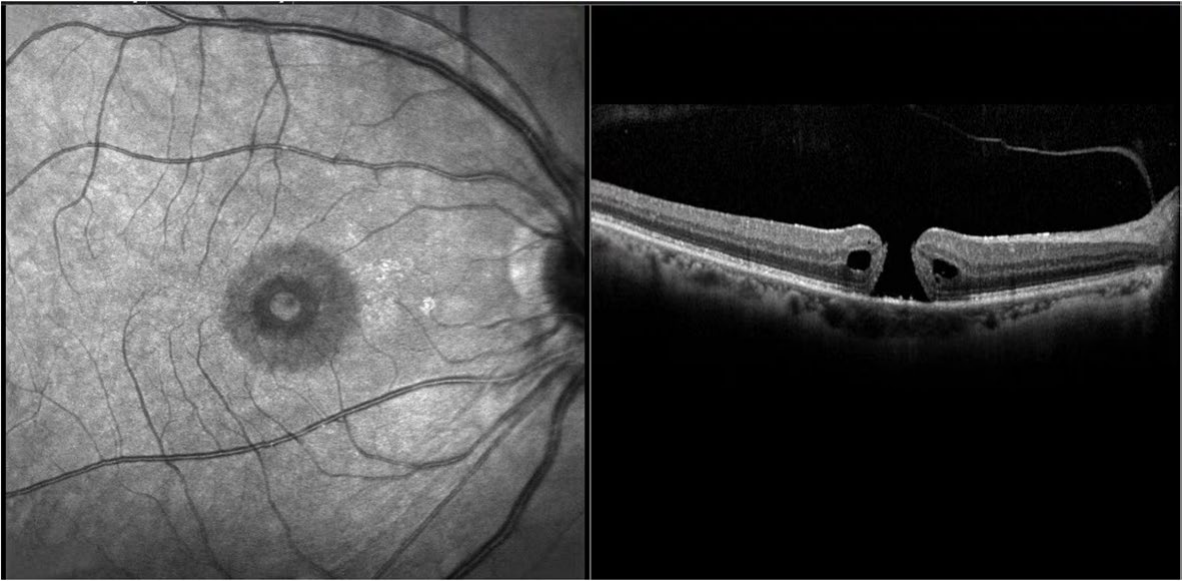
OCT of the right eye showed macular hole with cystic edema between layers

**Fig. 3:**
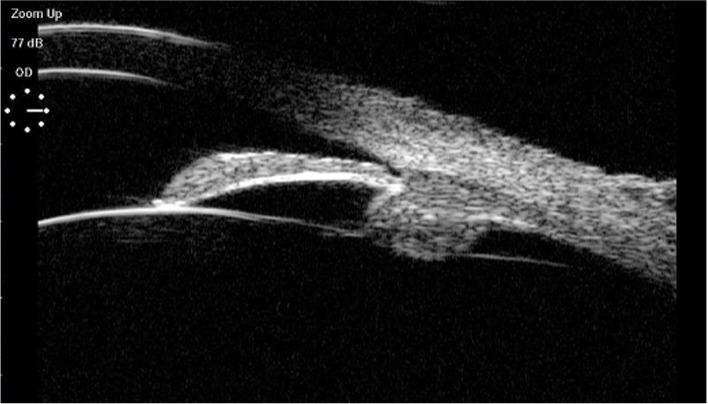
Ophthalmic ultrasound biomicroscopy showed the following ocular biometric findings: anterior lens position, anterior rotation of the ciliary body, shallow anterior chamber, and peripheral iris bombe of the right eye

Fundus examination revealed a right macular hole ($\frac{1}{4}$ pupillary distance) and a bilateral cup-to-disc ratio of 0.4 without signs of glaucomatous damage. The course and proportion of the bilateral retinal arteries and veins were normal. Optical coherence tomography showed complete loss of the neuroepithelial reflex in the right fovea(Fig. 2). Ophthalmic ultrasound biomicroscopy showed the following ocular biometric findings: anterior lens position, anterior rotation of ciliary body, shallowed anterior chamber, and peripheral iris bombe of the right eye (Fig. 3).

**Fig. 4:**
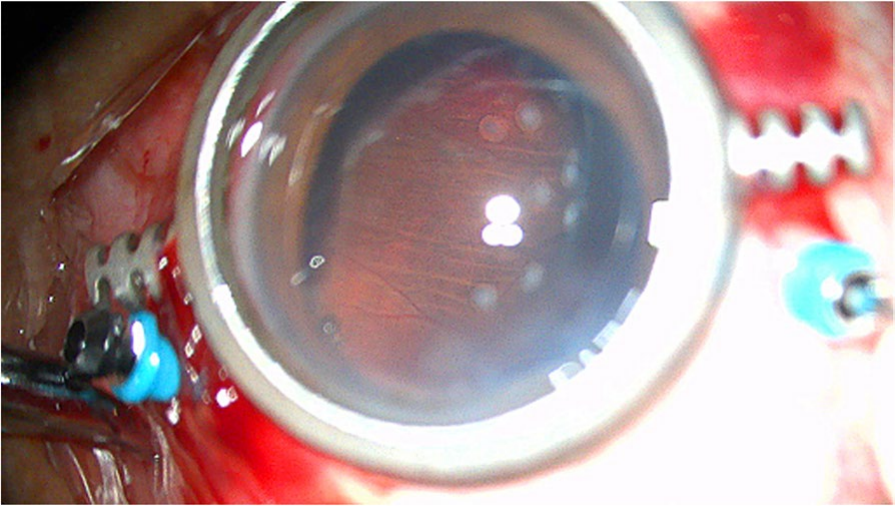
Many radial retinal stripes were found in the nasal peripheral retina during the surgery

We then performed vitrectomy combined with phacoemulsification and intraocular lens implantation surgery in the right eye for the diagnosis of macular hole and senile cataract (Additional file 1). Many pigmented, radially oriented lines on the anterior capsule (Fig. 1) were observed bilaterally and the zonule-free zone (ZFZ) was 2.5–3.0 mm wide. During continuous curvilinear capsulorhexis, it was found that the tension and toughness of the capsule were poor. At some of the zonules, the capsule began to tear radially along the fibers. When the peripheral vitreous was removed after posterior vitreous detachment, many radial retinal stripes were found in the nasal peripheral retina (Fig. 4). The left eye was carefully examined after the operation. In addition to anterior segment abnormalities similar to the right eye, several deep and shallow retinal stripes were also seen in the inferior and temporal sides of the peripheral retina. The intraocular pressure fluctuated from 19 to 22 mmHg in the three months following the operation.

## Discussion and conclusion

The anterior zonule usually ends on the anterior capsule 1.5 mm from the equator of the crystal. However, some patients have a more central zonular insertion zone, and a part more than 1 mm longer than normal is defined as an LAZ. LAZs are also noticed because of their obviously reduced ZFZ [3]. Since continuous curvilinear capsulorhexis was directed through lots of zonule’ insertions, the capsule is vulnerable to radially splitting along the fiber. Moreover, the risk of intraocular lens dislocation would be elevated [4, 5]. There are some methods reported in previously published literature that can avoid this capsular tear. First of all, the surgeon needs to keep the pupil edge out of the capsule edge during the process of capsulorhexis. The capsulorhexis forceps can be used to clamp the edge of the capsule flap and pull it towards the center of the pupil to change the direction of the anterior capsule tear to prevent peripheral extension of the tear [6, 7]. There is another way to rescue the torn anterior capsule in which the surgeon should unfold the capsulorhexis into its natural position, then grasp and pull back along the circumference, and finally pull inward towards the pupil center [8].

Because of the expansion of the contact area between the iris and zonules, the zonules become pigmented due to rubbing against the posterior iris pigment epithelium. There are also other pigment dispersal signs, such as Krukenberg spindles and trabecular meshwork pigmentation. These symptoms may cause Laz related chromatic dispersion syndrome or pigmentation glaucoma [9].

Except for open-angle glaucoma, there are few studies and case reports describing the existence of LAZs in angle-closure disease. Biological features of the eye play an important role in the pathogenesis of angle-closure disease. These include solitary relaxation, lens iris aperture mobility, curvature of the anterior lens surface, and lens arrangement disposed in front. Dysplastic zonules may also cause ciliary body anterior rotation and secondary angle closure [10]. Considering the clinical manifestations of this patient, the mechanism of high IOP is related to angle-closure disease.

The disease needs to be distinguished from congenital zonular relaxation, spherical lens, or Marfan syndrome because of the shallow anterior chamber, high IOP, and lens dislocation [11–13]. However, there are no obvious abnormalities in limb and heart development in patients with LAZs, and no iris tremor, obvious lens dislocation, or spherical changes have been found in patients with LAZs. The size of the ZFZ on the anterior lens capsule is obviously reduced, which is of great differential significance. However, if we do not carefully examine patients after mydriasis, LAZs may not be found, especially when they are not combined with other symptoms, which may be one of the reasons why LAZs are rarely reported.

Another phenotype has been reported in the literature, in which LAZs are associated with late-onset retinal and macular degeneration (L-ORMD). This is a rare disease characterized by night blindness in middle-aged individuals and decreased central and peripheral vision. Patients with L-ORMD have a S163R mutation in the *C1QTNF5* gene (C1q tumor necrosis factor-related protein 5), whereas the first phenotype has no known mutation [14].

These diseases of the anterior and posterior segments are related to early embryonic development. During embryonic development, the suspensory ligament is formed by the ciliary epithelium, and the Bruch membrane is formed by the retinal pigment epithelium (RPE). Both the ciliary epithelium and the RPE originate from the neuroectoderm. Both the suspensory ligament and the Bruch membrane contain glycoproteins. Therefore, the two diseases of LAZs and L-ORMD are linked [15].

The fundus features of L-ORMD include vitreous verrucous deposits, pigmented retinopathy, choroidal-retinal atrophy, choroidal neovascularization, and deposition between the retinal pigment epithelium and Bruch membrane. However, these fundus manifestations are not specific, and they are difficult to distinguish from other fundus diseases involving the outer retina and choroid, such as retinitis pigmentosa and age-related macular degeneration. If these fundus manifestations are combined with LAZ at the same time, the patient can be diagnosed with L-ORMD before genetic diagnosis [16]. In this case, there were both typical LAZs and a large amount of striped degeneration of retinal pigment deposition on one side of the peripheral retina. We determined this to be a case of LAZ complicated with peripheral retinal degeneration, which has not been reported before. However, this patient had the additional complication of a macular hole. There is no sufficient evidence to distinguish whether macular holes are idiopathic or caused by retinal degeneration in the outer layer of the macular region.

In conclusion, clinicians need to pay more attention to LAZs. Second, during capsulotomy, if the fiber is inevitably disconnected, it is necessary to increase the concern that the risk of radial capsule tear and intraocular lens dislocation may increase.Ttherefore, it is important to tear the appropriate size of the anterior capsule. Third, clinicians must be aware of both high IOP and RPE lesions in patients with LAZs. If necessary, gene detection and long-term observation should be carried out to improve our understanding of this disease.

## Supplementary Information

**Additional file 1.**

## Data Availability

The data used to support the findings of this study are available from the corresponding author upon request.

## Abbreviations

IOP: Intraocular pressure
LAZ: Long anterior lens zonule
RPE: Retinal pigment epithelium
L-ORMD: Late-onset retinal and macular degeneration
ZFZ: Zonule-free zone
